# Gut or Vein? Enteral Nutrition Versus Total Parenteral Nutrition in Acute Pancreatitis: A Systematic Review

**DOI:** 10.7759/cureus.108115

**Published:** 2026-05-01

**Authors:** Harnoor Kaur, Archit Sharma, Palangutde Katherine Dimowo, Akshay Rajeev, Sabah Shakeel Shaikh, Lubna Mohammed

**Affiliations:** 1 Internal Medicine, California Institute of Behavioral Neurosciences & Psychology, California, USA; 2 Paediatrics, Wrightington Wigan Leigh Hospitals NHS Foundation Trust, Wigan, GBR; 3 Otolaryngology - Head and Neck Surgery, California Institute of Behavioral Neurosciences & Psychology, California, USA; 4 Hematology, Great Western Hospital, Swindon, GBR; 5 Diabetes and Endocrinology, California Institute of Behavioral Neurosciences & Psychology, California, USA; 6 Principles and Practice of Clinical Research, Harvard School of Public Health, Boston, USA

**Keywords:** acute pancreatitis, acute pancreatitis complications, early enteral nutrition (een), enteral and parenteral nutrition, enteral nutrition (en), severe acute pancreatitis, total parenteral nutrition (tpn)

## Abstract

Acute pancreatitis is a common inflammatory condition with significant morbidity and mortality, particularly in patients who progress to moderately severe or severe disease. Nutritional support is an essential aspect of management, although the optimal route of nutrition remains debated. This systematic review assessed whether enteral nutrition (EN), compared with total parenteral nutrition (TPN), is associated with improved clinical outcomes in patients with acute pancreatitis. A thorough literature search was conducted across multiple electronic databases in accordance with Preferred Reporting Items for Systematic Reviews and Meta-Analyses (PRISMA) 2020 guidelines, including studies published between 1/01/2010 and 22/11/2025. A total of 1,480 records were identified, of which 1,382 records were screened after duplicate removal. Following title, abstract, and full text assessment, six studies met the predefined inclusion criteria and quality standards and were included in the final analysis. Eligible studies included human patients with acute pancreatitis that directly compared EN with TPN and reported clinically relevant outcomes such as morbidity and mortality. Study selection, data extraction, and quality appraisal were conducted using validated tools, including version 2 of* *A MeaSurement Tool to Assess systematic Reviews (AMSTAR 2) and the Newcastle-Ottawa scale. Across the included studies, EN was consistently associated with reduced mortality, fewer infectious complications, lower rates of multiple organ failure, and a reduced need for surgical intervention compared with TPN. Several studies also reported shorter hospital stays, although this finding was not uniform across all studies. Overall, the available evidence supports EN as the preferred nutritional strategy in acute pancreatitis, showing favorable effects on key clinical outcomes when compared with TPN. However, heterogeneity among studies remains high, highlighting the need for further research within contemporary clinical practice.

## Introduction and background

Acute pancreatitis is a prevalent hypermetabolic, hyperdynamic condition with a broad spectrum of severity, ranging from mild disease to severe acute pancreatitis, and arising from diverse etiologies [1,2]. Overall, the condition has a worldwide incidence of approximately 30-40 cases per 100,000 individuals annually, with rates exceeding double this figure in certain regions [3]. Gallstones and alcohol are still the most common causes of acute pancreatitis, although there are many other causes, such as metabolic, drug-induced, autoimmune, post-endoscopic retrograde cholangiopancreatography (ERCP), traumatic, infectious, genetic, congenital, and idiopathic [4]. The incidence of acute pancreatitis is also influenced by social and cultural factors, with an increase in alcohol-related cases in the United Kingdom during the Christmas and New Year weeks [5]. Approximately 20-30% of patients develop moderately severe or severe acute pancreatitis, which is associated with high morbidity and mortality rates, reaching up to 30% when complicated by infected pancreatic necrosis [6].

Severe acute pancreatitis (SAP) is marked by an increase in resting energy expenditure (REE). Patients with SAP experience significant metabolic stress, including increased protein breakdown and higher energy requirements, frequently resulting in undernutrition and clinically significant disturbances of fluid, electrolyte, and acid-base balance [7]. Nearly 30% of patients with SAP are already malnourished at presentation. Malnutrition further impairs wound healing and immune function, increasing the risk of infections and creating a vicious cycle of adverse clinical outcomes [8]. Therefore, adequate nutritional support is essential in patients with acute pancreatitis.

Nutrition management of acute pancreatitis has historically been guided by the principle of pancreatic rest to minimize exocrine pancreatic activation [9]. As a result, total parenteral nutrition (TPN) became the conventional modality for nutritional support in patients with SAP, as it permits delivery of exogenous nutrients while avoiding pancreatic secretory response [1]. TPN can help preserve lean body mass and prevent complications such as adynamic ileus [10]. However, bypassing the intestine as the main route of nutritional support can lead to loss of the mucosal barrier, which can intensify systemic inflammation and physiological stress, aggravating disease progression and increasing the risk of multiple organ failure, sepsis, and nosocomial infections [11]. Accumulating evidence suggests that TPN support is associated with an increased risk of infectious complications, as well as metabolic disturbances, including hyperglycemia and electrolyte imbalances, in addition to higher healthcare costs [1,8].

In contrast, enteral nutrition (EN) helps maintain intestinal barrier integrity, thereby reducing bacterial translocation [12,13]. Additionally, EN has also been associated with lower oxidative stress and reduced systemic endotoxin exposure, thereby suppressing the systemic inflammatory response, lowering the incidence of infectious complications, and reducing the healthcare cost [10,14]. Accordingly, the European Society for Clinical Nutrition and Metabolism (ESPEN) guidelines recommend the initiation of enteral nutrition within 24-72 hours of hospital admission in patients with SAP [15,16].

Although increasing evidence supports the use of EN in acute pancreatitis, its impact on clinically meaningful outcomes compared with TPN remains unclear. There is still a lot of uncertainty about how it will affect key endpoints like morbidity and mortality. Therefore, this systematic review aims to determine whether EN, in contrast to TPN, is linked to diminished morbidity and mortality in patients with acute pancreatitis.

## Review

Methods

This systematic review was carried out following Preferred Reporting Items for Systematic Reviews and Meta-Analyses (PRISMA) 2020 guidelines [17]. The research question and the review methods were predefined prior to conducting the review, and no significant deviations were made. This study was conducted as an interventional systematic review, comparing clinical outcomes between enteral nutrition and total parenteral nutrition. The review question was structured to evaluate the effectiveness of these interventions across the included studies. 

Database and Search Strategy

A comprehensive search was carried out across multiple databases, such as PubMed, using PubMed Medical Subject Headings (MeSH) and PubMed Advanced Search; Cochrane Library; ScienceDirect; Europe PMC; and Google Scholar. The core search strategy was formed using keywords like “acute pancreatitis,” “enteral nutrition,” and “total parenteral nutrition.” Only relevant literature from 1 January 2010 to 22 November 2025 was included in this review. Table 1 summarizes the database search strategies, including keywords, filters, and the number of records retrieved for each database.

**Table 1:** Summary of Search Strategies Used Across Various Databases and Number of Records Retrieved

Database	Keywords	Search strategy	Filters applied	Results
PubMed Advanced search	Acute pancreatitis, enteral nutrition, total parenteral nutrition	((Acute pancreatitis) AND (Enteral nutrition)) AND (Total Parenteral nutrition)	humans, free full text, English, studies between 01/01/2010- 22/11/2025	22
PubMed MeSH	Acute pancreatitis, enteral nutrition, total parenteral nutrition	ACUTE PANCREATITIS OR( "Pancreatitis/classification"[Mesh] OR "Pancreatitis/complications"[Mesh] OR "Pancreatitis/diet therapy"[Mesh] OR "Pancreatitis/mortality"[Mesh] OR "Pancreatitis/therapy"[Mesh] ) AND ENTERAL NUTRITION OR( "Enteral Nutrition/adverse effects"[Mesh] OR "Enteral Nutrition/classification"[Mesh] OR "Enteral Nutrition/methods"[Mesh] OR "Enteral Nutrition/mortality"[Mesh] OR "Enteral Nutrition/standards"[Mesh] OR "Enteral Nutrition/trends"[Mesh] ) AND TOTAL PARENTERAL NUTRITION OR ( "Parenteral Nutrition, Total/adverse effects"[Mesh] OR "Parenteral Nutrition, Total/classification"[Mesh] OR "Parenteral Nutrition, Total/methods"[Mesh] OR "Parenteral Nutrition, Total/mortality"[Mesh] OR "Parenteral Nutrition, Total/standards"[Mesh] OR "Parenteral Nutrition, Total/trends"[Mesh] )	humans, free full text, English, studies between 01/01/2010- 22/11/2025	234
Science Direct	Acute Pancreatitis, Enteral nutrition, Total parenteral nutrition, human	Acute pancreatitis, Enteral nutrition, Total parenteral nutrition, human	English, open access and open archive, review articles, research articles, studies between 01/01- 2010 – 22/11/2025	85
Cochrane Library	Acute pancreatitis, enteral nutrition, total parenteral nutrition, humans	acute pancreatitis in Title Abstract Keyword AND enteral nutrition in Title Abstract Keyword AND total parenteral nutrition in Title Abstract Keyword AND humans in Title Abstract Keyword	English, studies between 01/01/2010- 22/11/2025	17
Google Scholar	Acute pancreatitis, enteral nutrition, total parenteral nutrition, English, human	"acute pancreatitis" AND ("enteral nutrition" OR "enteral feeding") AND ("total parenteral nutrition" OR TPN) AND (morbidity OR mortality OR "clinical outcomes") AND (humans) AND (English)	Studies between 01/01/2010-22/11/2025	829
Europe PMC	Acute pancreatitis, enteral nutrition, total parenteral nutrition, English, human	(((((Acute pancreatitis) AND (enteral nutrition)) AND (Total parenteral nutrition)) AND (English)) AND (humans)) AND (((SRC: MED OR SRC: PMC OR SRC: AGR OR SRC:CBA) NOT (PUB_TYPE:"Review")) OR PUB_TYPE:REVIEW) AND (HAS_FT:Y) AND (FIRST_PDATE:[2010 TO 2025])	Studies between 01/01/2010-22/11/2025 Review articles, Research articles, Full text: In Europe PMC	293

Inclusion Criteria

Studies were considered eligible if they involved human patients with a confirmed diagnosis of acute pancreatitis and directly compared EN with TPN as defined intervention groups. Eligible study designs included systematic reviews, meta-analyses, comparative observational studies, and randomized controlled trials. Included studies were required to report at least one clinically meaningful outcome, specifically morbidity and/or mortality. Only full-text articles published or translated in the English language were included. 

Exclusion Criteria 

Studies were excluded if they focused on chronic pancreatitis, pancreatic malignancies, or postoperative nutritional support rather than acute pancreatitis. Also, studies that did not report relevant clinical outcomes were excluded. Animal studies or laboratory-based research studies, abstracts, unpublished work, narrative reviews, and case reports were also excluded from analysis.

Selection Process

Records identified through databases were imported to Rayyan AI for data management and duplicate removal. Records identified through Google Scholar and Europe PMC were initially managed in Mendeley and then imported to Rayyan AI. Titles and abstracts screening was conducted initially to exclude irrelevant articles. Full-text assessment was then performed for studies deemed potentially eligible, applying the predefined inclusion and exclusion criteria. Two reviewers independently screened titles, abstracts, and full texts for eligibility and extracted data. Any disagreements between reviewers were resolved through mutual discussion to achieve consensus. Finally, all qualifying studies were put through a structured quality appraisal to ensure methodological validity and look for any potential bias.

Quality Appraisal 

To ensure the reliability of the evidence, each study underwent quality appraisal using tools appropriate for the study design. Systematic reviews and meta-analyses were evaluated using version 2 of A MeaSurement Tool to Assess systematic Research (AMSTAR 2) [18], with careful attention to critical domains. Studies were included if they had no more than one weakness in critical domains and performed well in other areas, such as risk of bias assessment, methods, and comprehensiveness of the literature search. Additionally, comparative observational studies were appraised using the Newcastle-Ottawa scale [19], ensuring systematic assessment of study quality and bias. Risk of bias and quality were independently assessed by two reviewers using design-specific appraisal tools. Any disagreements were resolved through discussion between the reviewers.

Data Synthesis 

Due to heterogeneity in study design, populations, and outcome reporting among included studies, a meta-analysis was not performed, and findings were synthesized using a qualitative (narrative) approach.

Results

**Figure 1:**
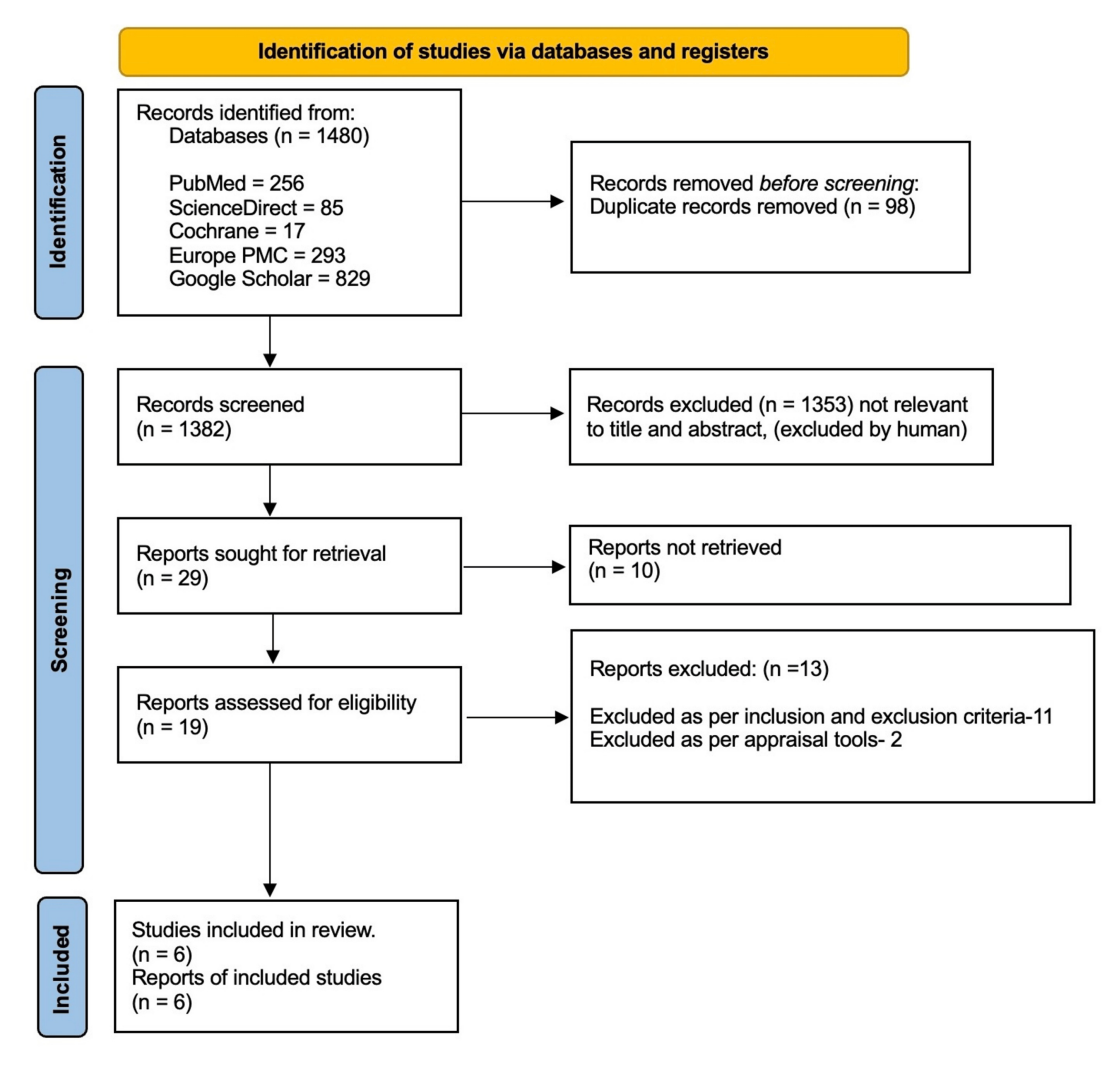
PRISMA 2020 Flow Diagram for Systematic Reviews Illustrating Study Selection Process PRISMA- Preferred Reporting Items for Systematic Reviews and Meta-Analyses [17]

A total of 1,480 records (PubMed: 256; ScienceDirect: 85; Cochrane: 17; Europe PMC: 293; Google Scholar: 829) were identified across all databases. After duplicate removal, 1382 records underwent title and abstract screening, of which 1353 were excluded. A total of 29 studies were sought for retrieval, of which 10 could not be retrieved. The remaining 19 reports underwent full-text assessment. Of these, 11 studies were excluded based on predefined inclusion and exclusion criteria. The remaining eight studies proceeded to quality appraisal using design-appropriate tools, following which two studies were excluded due to methodological limitations. Ultimately, six studies were included in the final systematic review. Although randomized controlled trials were considered eligible, none met the inclusion criteria following screening, and therefore none were included in the final analysis. Two of the included studies showed weakness in one critical domain (Domain 2: pre-registered protocol) of the AMSTAR 2 tool. However, both performed well in all other domains and demonstrated consistent findings; hence, they were included in the review. This limitation is acknowledged in the discussion section. The results of the study selection process are presented in the PRISMA 2020 flow diagram in Figure 1.

The methodological quality of the included systematic reviews and meta-analyses was assessed using the AMSTAR-2 tool [18], with the results summarized in Table 2.

**Table 2:** Methodological Assessment of Included Systematic Reviews and Meta-Analyses Using the AMSTAR 2 Tool Study quality of systematic reviews and meta-analyses was assessed using the AMSTAR 2 tool. Each item was rated as 'Yes,' 'Partial Yes,' or 'No' in line with AMSTAR 2 guidance, with 'Partial Yes' applied only where permitted. Critical domains were evaluated and interpreted according to AMSTAR 2 recommendations [18]. PICO: population, intervention, comparison, outcome; RoB: risk of bias.

	AMSTAR 2 Domain	Al-Omran et al. [1]	Wu et al. [2]	Li et al. [10]	Hsieh et al. [20]	Liu et al. [8]
1.	PICO clearly defined	Yes	Yes	Yes	Yes	Yes
2.	Protocol registered prior to review	Yes	No	No	Yes	Partial Yes
3.	Rationale for study design selection	Yes	Yes	Yes	Yes	No
4.	Comprehensive literature search	Yes	Partial Yes	Partial Yes	Yes	Partial Yes
5.	Study selection in duplicate	Yes	Yes	Yes	Yes	Yes
6.	Data extraction in duplicate	Yes	Yes	Yes	Yes	Yes
7.	Excluded studies listed with justification	Yes	Partial Yes	Partial Yes	Partial Yes	Partial Yes
8.	Adequate description of included studies	Yes	Yes	Yes	Yes	Yes
9.	Appropriate risk of bias assessment	Yes	Yes	Partial Yes	Yes	Yes
10.	Funding sources of included studies reported	Yes	No	No	No	No
11.	Appropriate meta-analysis methods	Yes	Yes	Yes	Yes	Yes
12.	Impact of RoB on results assessed	Yes	No	No	Yes	Yes
13.	RoB considered in interpretation	Yes	Yes	Yes	Yes	Yes
14.	Heterogeneity assessed and discussed	Yes	Yes	Yes	Yes	Yes
15.	Publication bias assessed	No	Yes	Yes	Yes	Yes
16.	Conflict of interest reported	Yes	Yes	Yes	Yes	Yes

The quality appraisal of the included observational study was conducted using the Newcastle-Ottawa Scale [19], with the assessment presented in Table 3.

**Table 3:** Quality Appraisal of Included Study Using Newcastle-Ottawa Quality Assessment Scale In accordance with the Newcastle-Ottawa quality assessment scale for cohort study, each item within the selection and outcome domains was awarded a maximum of one star, while up to two stars could be assigned for the comparability domain. Newcastle-Ottawa Scale domains: Selection (Q1: representativeness of the exposed cohort; Q2: selection of the non-exposed cohort; Q3: ascertainment of exposure; Q4: demonstration that outcome of interest was not present was not present at start of study); Comparability (Q5: comparability of cohorts based on the design or analysis); Outcome: (Q6: assessment of outcome; Q7: was follow-up long enough for outcomes to occur; Q8: adequacy of follow up of cohorts) [19].

Authors	Study type	Selection				Comparability	Outcome			Total stars	Quality
		Q1	Q2	Q3	Q4	Q5	Q6	Q7	Q8		
Vieira et al. [11]	Retrospective, cohort study	*	*	*	*	*	*	*	*	8/9	High

Discussion

Nutritional Strategies in Acute Pancreatitis

This systematic review brings together evidence from six studies comparing EN with TPN in patients with acute pancreatitis. Overall, the included literature consistently shows that EN is associated with improved clinical outcomes, including reduced mortality, fewer infectious complications, lower rates of multiple organ failure (MOF), and a reduced need for surgical intervention. Several studies also reported shorter hospital stays with EN, although this finding was less consistent across study designs. Collectively, these findings point towards EN as the preferred nutritional strategy in acute pancreatitis, particularly in patients with severe disease [1,2,8,10,11,20]. These findings are consistent with contemporary ESPEN guidelines, which recommend EN as the preferred route of nutritional support in acute pancreatitis and support early initiation to improve clinical outcomes when oral feeding is not tolerated [15]. Parenteral nutrition should be reserved for patients with acute pancreatitis only when oral and enteral feeding are not feasible, not tolerated, or insufficient to meet nutritional requirements [6,9,14,16]. Table 4 presents a summary of the key characteristics and findings of the six included studies, including study design, sample size, disease severity, and reported clinical outcomes.

**Table 4:** Characteristics and Key Findings of Included Studies Comparing EN Versus TPN in Patients with Acute Pancreatitis ICU: intensive care unit; EN: enteral nutrition; RCTs: randomized controlled trials; MODS: multiple organ dysfunction syndrome; TPN: total parenteral nutrition; APACHE II: acute physiology and chronic health evaluation II.

Author, Year	Study design	Sample size	Study Population	Severity of acute pancreatitis	Outcomes assessed	Key findings
Vieira et al. [11]	Retrospective observational study	31	Hospitalized adult ICU patients with acute pancreatitis	Severe	Mortality, septic complications, length of stay	EN resulted in fewer septic complications, reduced mortality, and shorter hospital stay
Al-Omran et al. [1]	Systematic review and meta- analysis	348	Hospitalized adult patients with acute pancreatitis (RCTs)	Predominantly severe	Mortality, infections, need for surgery, organ failure	EN is associated with lower mortality, fewer infections, reduced surgical interventions and organ failure
Wu et al. [2]	Meta-analysis	562	Hospitalized adult patients with acute pancreatitis (RCTs and cohort studies)	Severe	Mortality, infection rate, MODS	EN significantly reduced mortality, infection rates, and incidence of MODS
Li et al. [10]	Meta-analysis	500	Hospitalized adult patients with acute pancreatitis (RCTs)	Severe	Mortality, infectious complications, ICU stay, hospital stay	EN reduced mortality, infection rates, ICU length of stay, and overall hospital stay
Hsieh et al. [20]	Systematic review with network meta- analysis	16 RCTs	Hospitalized adult patients with acute pancreatitis (RCTs)	Mild- severe	Infection rate	EN is associated with significantly lower infection rates compared with TPN
Liu et al. [8]	Systematic review and meta-analysis	699	Hospitalized adult patients with acute pancreatitis (RCTs ; APACHE II >6)	Severe	Mortality, infectious complications, organ failure, length of stay	EN significantly reduces mortality, infections, multiple organ failure, and hospital stay compared with TPN

Acute Pancreatitis- Diagnosis and Severity 

Acute pancreatitis is an inflammatory condition with a broad clinical spectrum, ranging from a mild, self-limiting form characterized by interstitial pancreatic edema to a severe manifestation associated with extensive pancreatic necrosis [21]. A definitive diagnosis of acute pancreatitis requires the presence of at least two of the following three criteria: abdominal pain typical of acute pancreatitis; serum amylase and/or lipase levels elevated to three times or more the upper limit of normal; characteristic findings of acute pancreatitis on cross-sectional imaging, such as computed tomography (CT) or magnetic resonance imaging (MRI), or occasionally transabdominal ultrasound (TUS) [3].

Given the highly variable clinical course of acute pancreatitis, early severity stratification using scoring systems such as Acute Physiology and Chronic Health Evaluation (APACHE II) and Ranson’s criteria, Glasgow, Bedside Index for Severity in Acute Pancreatitis (BISAP), and Systemic Inflammatory Response Syndrome (SIRS) is important to identify patients at risk of complications and for guiding management decisions [8,22]. Most included studies have trials that classified patients as having severe or predicted SAP based on clinical criteria predefined by the original study authors [1,2,6,10,11].

Across the included studies, severity assessment was defined using a combination of clinical scoring systems and radiological criteria, most commonly the APACHE II score and Ranson’s criteria [1,4,8], as well as contrast-enhanced CT-based assessment of pancreatic and peripancreatic abnormalities [2]. In one study, inclusion was restricted to patients classified as Balthazar grades C-E, consistent with SAP [11].

Gut-Pancreas Axis and the Impact of Nutritional Strategies on Infectious Outcomes in Acute Pancreatitis

**Figure 2:**
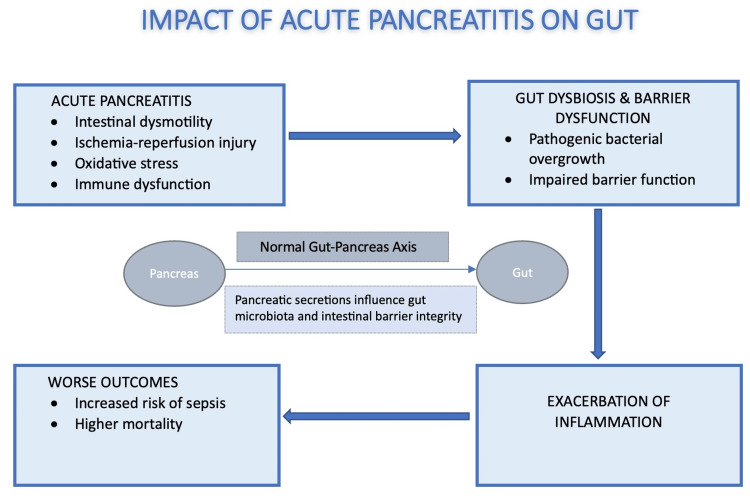
Impact of Acute Pancreatitis on Gut-Pancreas Axis and Further Consequences This image was created by first author using Microsoft Word based on data from [23,24].

The gut-pancreas axis plays an important role in maintaining host homeostasis, as pancreatic secretions influence intestinal microbial composition and gut barrier integrity. In acute pancreatitis, gut microbial dysbiosis may result from intestinal dysmotility, ischemia-reperfusion injury, oxidative stress, and immune dysfunction [23,24]. These processes promote pathogenic bacterial overgrowth, disruption of the epithelial barrier, and worsening of inflammation [23,24]. Figure 2 illustrates the proposed mechanisms linking gut dysbiosis and pancreatic inflammation, although it remains uncertain whether gut dysbiosis is a cause or consequence of pancreatic injury [23,24].

All six studies included in this review highlight the central role of the intestinal barrier in progression and complications of acute pancreatitis and its distinct modulation by EN as compared to TPN [1,2,8,10,11,20]. Across all studies, EN is consistently described as preserving intestinal mucosal integrity and barrier function, whereas TPN bypasses the gastrointestinal tract and fails to provide trophic stimulation to the intestinal mucosa [1,2,8,10,11,20]. This absence of luminal nutrient exposure during TPN has been associated with mucosal atrophy and increased intestinal permeability, facilitating translocation of enteric bacteria and endotoxins into the systemic circulation and pancreatic tissue, thereby contributing to infected pancreatic necrosis and sepsis [2,8,11,20].

These mechanisms are reflected in the clinical findings reported in the included studies. In studies comparing EN with TPN, infectious complications, including catheter-related sepsis, pancreatic and peripancreatic infections, bacteremia, and pneumonia, were more commonly reported in patients receiving TPN [11,20]. In contrast, EN was consistently associated with lower infection rates and fewer septic complications [1,11,20]. Comparative evidence also demonstrated a reduced rate of infected pancreatic necrosis and overall infectious morbidity with EN compared with TPN, supporting the protective role of maintaining gut barrier integrity [2,20]. In addition, EN has been linked with lower serum endotoxin levels, improved markers of intestinal permeability, and reduced systemic inflammatory response [2,8,10,11]. Hsieh et al. (2019) [20] described this as a shift in the management of acute pancreatitis, from prioritizing pancreatic rest to preserving gut barrier integrity and preventing bacterial translocation, supporting EN as first-line therapy.

Influence of Nutritional Strategies on Mortality and Organ Dysfunction

**Figure 3:**
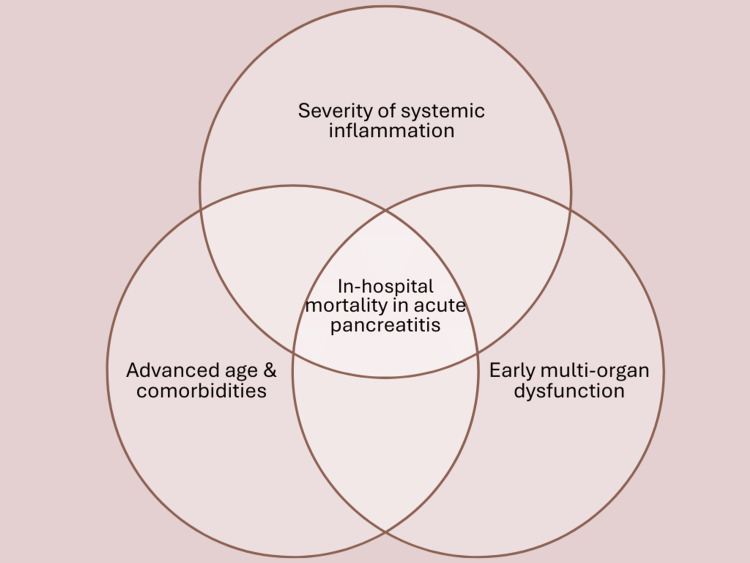
Factors Affecting In-Hospital Mortality of Patients with Acute Pancreatitis This figure was created by first author using Canva (Canva Pty Ltd, Australia) based on data from [25].

In-hospital mortality in acute pancreatitis is influenced by a combination of systemic and patient-related factors rather than pancreatic injury alone. Figure 3 illustrates the key contributors, including severity of systemic inflammation, advanced age and comorbidities, and early multi-organ dysfunction, particularly respiratory and renal failure [25].

In the included studies, EN showed a protective trend against MOF compared with TPN, although the strength of evidence varied depending on study design and outcome definition [1,2,8,10,11,20]. The Cochrane review reported a statistically significant reduction in MOF with EN, supporting a meaningful clinical benefit in preventing systemic deterioration [1]. Both Liu et al. [8] and Wu et al. [2] reported lower MOF rates with EN that did not reach statistical significance. While Hsieh et al. [20] did not assess MOF directly, the observed reductions in infected pancreatic necrosis and systemic infection with EN suggest an indirect protective effect against progression to organ failure. Finally, Vieira et al. [11] reported MOF occurring only in the TPN group, linking organ failure to septic complications.

Consistent with the observed effects on MOF, all studies that reported mortality outcomes demonstrated a reduction in mortality associated with EN compared with TPN [1,2,8,10,11]. Al-Omran et al. (2010) [1] demonstrated a significant mortality reduction with EN, particularly in SAP. Similarly, Wu et al. [2] and Li et al. [10] also reported consistent survival benefits with low heterogeneity, strengthening the evidence for improved mortality outcomes. Liu et al. [8] similarly observed statistically significant mortality reduction with EN, although the effect size was smaller, suggesting potential variability related to baseline risk and study inclusion criteria. In contrast, Hsieh et al. [20] did not directly assess mortality but demonstrated marked reductions in infectious complications known to precipitate organ failure and death [20]. Finally, Vieira et al. [11], despite limited power, reported all deaths occurring exclusively in the TPN group.

Effect of Nutritional Strategies on Clinical Course and Resource Utilization 

Beyond its effect on MOF and survival, nutritional strategies also influence clinical course and healthcare resource utilization in acute pancreatitis. Across the included studies, EN was generally associated with a more favorable recovery than TPN, although effects varied by outcome and study design [1,2,8,10,11,20]. Meta-analyses by Liu et al. [8] and Wu et al. [2] demonstrated a significantly shorter length of hospital stay with EN, suggesting faster clinical stabilization, whereas Al-Omran et al. [1] reported a non-significant trend that was more evident in SAP subgroups. In contrast, Li et al. (2018) [10] and Vieira et al. (2010) [11] did not observe any statistically significant reductions in hospital stay.

In comparison, the requirement for surgical intervention showed a more consistent and clinically meaningful reduction with EN [1,10,11,20]. A Cochrane review and later meta-analyses reported a significant decrease in operative intervention with EN, particularly in severe disease, reflecting lower rates of complication-driven surgery [1,2,10]. Notably, Vieira et al. (2010) [11] reported that pancreatitis-related surgeries, including necrostomy, occurred exclusively in the TPN group, while procedures in the EN group were limited to elective biliary intervention. Although Hsieh et al. [20] did not directly assess hospital stay or surgery, their demonstration of reduced infected pancreatic necrosis and improved feasibility of NG feeding provides indirect support for reduced downstream resource utilization.

Limitations

This systematic review has several limitations. A key limitation is the presence of clinical and methodological heterogeneity among the included studies, particularly in study design, patient populations (including variations in disease severity and clinical characteristics), and outcome reporting, which limited the feasibility of quantitative pooling. Secondary outcomes such as the length of hospital stay and surgical intervention were inconsistently reported, and some conclusions relied on indirect evidence rather than direct measurement. Additionally, several studies were conducted in earlier eras of pancreatitis management, potentially limiting generalizability to contemporary practice. There was also a lack of uniformity in EN protocols, including differences in formulation, route, and timing of initiation, which may have influenced outcomes. Notably, two of the included reviews had a weakness in one AMSTAR 2 domain related to protocol registration. Although they performed satisfactorily in all other important methodological aspects, this is recognized as a limitation. Finally, the possibility of publication bias should also be acknowledged.

## Conclusions

This systematic review integrates available evidence on nutritional strategies in acute pancreatitis and demonstrates that EN is consistently associated with superior clinical outcomes compared with TPN. Across the included studies, EN was linked to reduced mortality, fewer infectious complications, and a lower progression to organ dysfunction, particularly in patients with severe acute pancreatitis, supporting its role as the preferred route of nutritional support. The observed clinical benefits of EN are supported by a strong underlying physiological basis, primarily through preservation of gut barrier integrity, attenuation of systemic inflammation, and reduction in bacterial translocation, which together mitigate the development of sepsis and multi-organ failure. Clinically, these findings support early initiation of EN as the standard of care in patients with acute pancreatitis whenever feasible and not contraindicated. Despite consistent signals of benefit, heterogeneity in study designs, nutritional protocols, and outcome definitions limits direct comparability and generalizability. Future well-designed randomized controlled trials are needed to better standardize severity stratification, timing, and composition of enteral feeding and to evaluate patient-centered outcomes in order to further refine nutritional guidelines in acute pancreatitis.
